# Neurobiological Correlates of Rheumatoid Arthritis and Osteoarthritis: Remodelling and Plasticity of Nociceptive and Autonomic Innervations in Synovial Joints

**DOI:** 10.1177/10738584241293049

**Published:** 2024-12-12

**Authors:** Sharon Mathew, Sadaf Ashraf, Susan Shorter, Gianluca Tozzi, Stella Koutsikou, Saak V. Ovsepian

**Affiliations:** 1 Faculty of Engineering and Science, University of Greenwich London, Chatham Maritime, Kent, UK; 2 Medway School of Pharmacy, Universities of Kent and Greenwich, Chatham Maritime, Kent, UK; 3 Faculty of Medicine, Tbilisi State University, Tbilisi, Republic of Georgia

**Keywords:** rheumatoid arthritis, neuroinflammation, chronic pain, sensitization, silent nociceptors, remodelling and neuroplasticity

## Abstract

Swelling, stiffness, and pain in synovial joints are primary hallmarks of osteoarthritis and rheumatoid arthritis. Hyperactivity of nociceptors and excessive release of inflammatory factors and pain mediators play a crucial role, with emerging data suggesting extensive remodelling and plasticity of joint innervations. Herein, we review structural, functional, and molecular alterations in sensory and autonomic axons wiring arthritic joints and revisit mechanisms implicated in the sensitization of nociceptors, leading to chronic pain. Sprouting and reorganization of sensory and autonomic fibers with the invasion of ectopic branches into surrounding inflamed tissues are associated with the upregulation of pain markers. These changes are frequently complemented by a phenotypic switch of sensory and autonomic profiles and activation of silent axons, inferring homeostatic adjustments and reprogramming of innervations. Identifying critical molecular players and neurobiological mechanisms underpinning the rewiring and sensitization of joints is likely to elucidate causatives of neuroinflammation and chronic pain, assisting in finding new therapeutic targets and opportunities for interventions.

## Introduction

Arthritis is a heterogeneous group of diseases affecting synovial joints, causing persistent pain, stiffness, and inflammation, which could lead to lifetime disability. There are more than 100 types of arthritis, with osteoarthritis (OA) and rheumatoid arthritis (RA) as the most prevalent forms (Di Matteo and others 2023; Ding and others 2024; Smolen and others 2018). Over one-third of adults in Western countries have some degree of arthritis, with OA accounting for over ~70% of cases, followed by the second most common, RA (Ding and others 2024; Smolen and others 2018). Despite differences in etiology and pathophysiological mechanisms, OA and RA share clinical signs of painful and swollen joints caused by underlying inflammation and tissue damage. In the case of OA, the primary cause is the “wear and tear” of the joint cartilage and synovium, making the condition more common among the aging and overweight population. OA changes are also occasionally seen following traumatic joint injury (Curtis and others 2016; Raterman and others 2020). On the other hand, RA is an autoimmune condition where the immune system attacks joint tissues, especially the synovium (Guo and others 2018; Smolen and others 2018). Depending on the type and severity of arthritis, the pathophysiology may vary from mild to intermittent stiffness and joint pain to severe and progressive erosion of the articular tissue, leading to permanent damage and disability.

The peripheral mechanisms of arthritic pain are associated to a large degree with sensitization and hyperactivity of joint innervations (Eitner and others 2017; Miyamoto and others 2021; Thakur and others 2014). Swelling and tenderness of synovial joints in RA and OA are accompanied by the invasion of immune cells and local neuroinflammation, causing tissue breakdown with the formation of pannus and disruption of the synovial lining (Cao and others 2020; Guo and others 2018; Molnar and others 2021; Smolen and others 2018). The progression of the disease and related inflammation leads to persistent and unrelenting pain, which, together with stiffness, hinders the functions of affected joints (George and others 2020; Huang and others 2021; Koenders and others 2015; Yao and others 2023). Densely innervated by sensory and autonomic nerves, arthritic joints undergo a drastic neurochemical and immunological stress contributed by the release of a cocktail of transmitters, trophic factors, and mediators of inflammation. These alterations lead to sensitization and hyperactivity of nociceptors, exacerbating the neuroinflammation and chronic pain (Eitner and others 2017; Eitner and others 2013; Fingleton and others 2015; Zhang and Lee 2018).

Despite impressive therapeutic advances, there is a substantial unmet need in the management of pain associated with OA and RA, which presents the main reason for seeking medical assistance. Reducing local inflammation and pain relief with nonsteroidal anti-inflammatory drugs (NSAIDs) and painkillers, as well as slowing disease progression with antirheumatic medications, are the first-line therapies (Bullock and others 2018; Kloppenburg 2024; Latourte and others 2020). Failure of pharmacological treatments in a significant fraction of patients necessitates invasive surgery with joint replacement (Evans and others 2022; Evans and others 2019). The lack of a radical cure for OA and RA with a variety of side effects of current therapies warrants mechanistic studies to identify new targets for more effective and personalized interventions. To this end, research on neural remodelling with sensitization and plasticity of nociceptors in affected tissue can be highly instructive. Although sensitization of nociceptors is viewed as the primary peripheral mechanism of chronic pain (McDougall 2006; Ovsepian and Waxman 2023; Zheng and others 2022), growing data also suggests the importance of remodelling of nociceptive and autonomic innervations of joints with the ectopic activity of pain-sensing nerve fibers. Mounting histopathological evidence also implies a phenotypic switch of axons and activation of “silent” nociceptors, suggestive of reprogramming of sensory and autonomic neurons and regulatory changes (Obeidat and others 2023; Stangl and others 2015).

In this review, we present preclinical and clinical evidence for extensive structural and molecular remodelling of sensory and autonomic innervations in OA and RA joints. We consider the putative molecular mechanism and discuss homeostatic and regulatory adjustments contributing to inflammation and sensitization of joint nociceptors. Described herein alterations not only provide insights into the neurobiological mechanisms of pain in joints of OA and RA but also suggest an array of new targets for therapeutic interventions to improve the management of these prevalent conditions.

## Methods and Data Presentation

All the authors conducted a literature search of OA and RA using scientific databases such as PubMed and ScienceDirect. Where necessary, Google Scholar, Academia, and Research Gate have been used as additional sources of information and references. The following keywords were used for the literature search: rheumatoid arthritis and joint pain, osteoarthritis and joint pain, alterations in innervations of arthritic joints, sensory remodelling of arthritic joints, arthritis and neuroplasticity, axonal sprouting and arthritis pain, joint nociceptive sensitization, nociceptors of joints in arthritis, sympathetic innervation of joints in arthritis, parasympathetic innervation of joints in arthritis, autonomic innervation of joints, inflamed and painful joints in arthritis, mechanisms of arthritic pain; rheumatoid arthritis and pain, and osteoarthritis and pain. The reference list of articles was scanned to identify information relevant to the current analysis. All papers reviewed in this article have been scrutinized and selected based on (1) scientific rigour of presented data with carefully designed experiments and reference to controls, (2) adequate sample size with a number of replicas and variability, (3) valid statistical analysis and test with the significance of *P* values, and (4) data quality and reproducibility, with discussions limited to the peer-reviewed papers only (Snyder 2019; Yu and others 2017). A summary of all references was drafted, followed by thematic grouping and manuscript writing. Figures have been prepared using Adobe Illustrator Artwork 16.0 of the Adobe Creative Suit version 6.0. Tables have been generated using Microsoft Word. EndNote 21 was used for citations, with references formatted per journal guidelines.

## Anatomy and Innervation of Synovial Joint: A Brief Overview

Known also as diarthrosis, a synovial joint connects two bones with a fibrous capsule, which is continuous with the periosteum of the bones. The capsule forms the outer scaffold of the joint cavity, providing mechanical connection and supporting bone movement (Martini and others 2018). It also seals the synovial chamber filled with fluid, which prevents friction between the articular cartilages covering the joint surface of connecting bones (Juneja and others 2018). Lining the inner surface of the articular capsule is the synovial membrane, with cells producing the synovial fluid. Fibrous ligaments connecting bones allow for regular movements at a joint but limit their range to specific functions (Martini and others 2018; Tamer 2013). Ligaments of synovial joints are classified as extrinsic, located outside of the articular capsule, fused to the wall of the capsule, and intracapsular, embedded inside the capsule.

The innervation of healthy synovial joints is organized in line with two principles. The first is Gardner’s principle, which holds that the part of the articular capsule that is tightened by contraction of a group of muscles receives a nerve supply from nerves that innervate the antagonist muscles. This arrangement enables stabilizing effects of local reflex arcs on the joint. The second, Hilton’s principle, maintains that the sensory fibers innervating articular capsule and ligaments are branches of the nerves that supply muscles responsible for moving it (Juneja and others 2018). Therefore, irritation of articular nerves causes a reflector spasm of the muscles, which position the joint for the highest comfort. These nerves also supply fibers to the overlying dermal tissue, relating the nociception of the joint with the skin.

**Figure 1.**
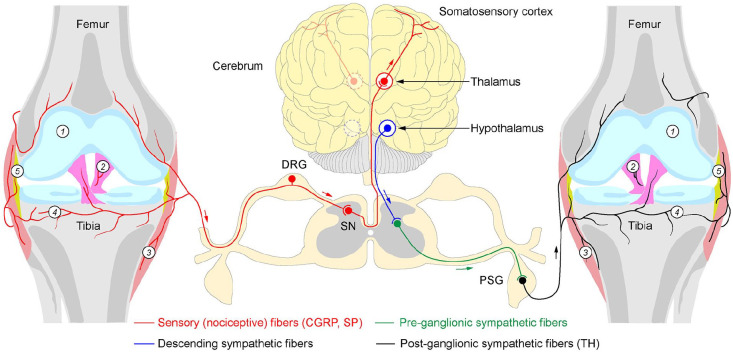
Sensory and autonomic innervation in a healthy human synovial joint. The human knee joint receives proprioceptive and nociceptive innervations from the dorsal root ganglion neurons (DRG), which convey primary sensory signals to the dorsal horn of the spinal cord (spinal neurons, SN). From there, information propagates to thalamic neurons, which transmit signals to the somatosensory cortex. The human joint also receives autonomic innervations from paravertebral sympathetic neurons (paravertebral sympathetic ganglion, PSG, black), which are under direct modulation of preganglionic neurons of the spinal cord (green), receiving descending inputs from the hypothalamus (blue). Note the innervation-free cartilage of the healthy joint (1). Nociceptive, calcitonin gene–related peptide (CGRP), substance P (SP) immunoreactive and autonomic, tyrosine hydroxylase (TH) immunoreactive axons innervate various compartments of the knee joint tissues, including ligaments (2), joint capsule (3), subcartilaginous bone (4), and synovium (5). Note that no parasympathetic innervation is detectable in healthy joints. Red arrow: centripetal flow of sensory signals; green and black arrows: centrifugal flow of autonomic signals.

All synovial joints receive sensory and autonomic innervations (Fig. 1). The autonomic innervations comprise exclusively sympathetic postganglionic nerve fibers, which are reactive to tyrosine hydroxylase (TH) and vesicular monoamine transporter 2 (VMAT-2) antibodies and release norepinephrine (NE). The autonomic nerves regulate vasomotor functions, controlling the supply of nutrients and oxygen to the joint (Halata and others 1985; Juneja and others 2018). The healthy synovial joints lack cholinergic innervations, as evident from the absence of vesicular acetylcholine transporter (VAChT) and vasoactive intestinal protein (VIP) immunoreactive profiles (Courties and others 2020; Courties and others 2017). Sensory innervations of synovial joints, on the other hand, are mediated by two types of fibers: nociceptive and proprioceptive. Light and electron microscopy studies have shown that most nerves of synovial joints are nociceptive (sensory A-δ and C fibers), innervating the capsule, synovium, ligaments, menisci, periosteum, and subchondral bone (Fig. 1). They are immunoreactive to calcitonin gene–related peptide (CGRP) and substance P (SP). The remaining sensory innervations are of neurofilament 200 (NF200)–positive thick proprioceptive A-β fibers with Ruffini-, Golgi- and Pacini-type endings in ligaments, fibrous capsules, menisci, and adjacent periosteum (Obeidat and others 2019). Functionally, fast-conducting thick proprioceptive axons support the sense of posture, locomotion, and movement, while thin free nerve endings of nociceptive axons of the joint convey the sensation of diffuse and poorly localized pain (Halata and others 1985; Juneja and others 2018). Although the overall organization of sensory and autonomic innervations of healthy synovial joints is well established, as illustrated in the following, their changes in OA and RA are a subject of controversy and in-depth studies.

## Remodelling Sensory Innervations in Inflamed Synovial Joints of OA and RA

### Clinical Studies

Research suggests extensive structural and molecular alterations in the sensory innervation of arthritic joints via gain or loss of axonal branches and changes in their neurochemical profiles (Table 1). In the human OA knee joint, remodelling of nociceptive innervation generally correlates with the extent of inflammation, with reports suggesting an invasion of new axonal sprouts into the synovial tissue (Eitner and others 2013). In clinical OA samples, nerve fibers of the joint were observed in deeper synovial layers than in healthy, with their density correlating with the synovitis score. Notably, the severely inflamed synovium regions of OA joints showed aberrations of capillary networks and loss of nerve fibers (Eitner and others 2013). Focal reduction of the innervation in strongly inflamed areas of synovial joints has also been observed in RA (Eitner and others 2013). Histochemical analysis revealed lower density of SP immunoreactive profiles in OA compared to RA or control synovial tissues (Weidler and others 2005). Notably, in OA joints, the density of SP-positive nociceptive fibers correlates with that of TH-positive sympathetic axons, with the latter also degenerating in severely affected areas (Weidler and others 2005). Contrasting to healthy joints with unmyelinated axons arranged along the blood vessels and showing specific nociceptor markers, in OA, nerve sprouts spread over articular cartilage and express sprouting-specific protein gene product (PGP)–9.5 in equal amounts in sensory (SP- and CGRP-positive) and sympathetic (C-terminal flanking peptide of neuropeptide Y–NPY, CPON-positive) fibers (Suri and others 2007).

**Table 1.** Alterations in Sensory Innervation of Arthritic Joints in Human Studies and Preclinical Models, with Analyzed Joints, Specific Changes in Innervations, Functional Readouts, and References.

Condition	Model	Joint	Axonal Markers	Remodelling and Plasticity	Readouts	References
OA	Rat	Ankle joint	CGRP	Sprouting and entwining with sympathetic axons	Pain, hypersensitivity	(Longo and others 2013)
OA	Rat	Ankle joint	CGRP, NF200	Sprouting, sensitization by inflammatory factors	Pain	(La Hausse De Lalouviere and others 2021)
OA	Mouse	Knee joint	CGRP, GAP43, NF200	Ramified and diffuse sprouting	Pain	(Ghilardi and others 2012)
OA	Mouse	Knee joint	CGRP, GAP43, NF200	Ramified and diffuse sprouting, formation of neuroma-like elements	Pain	(Jimenez-Andrade and Mantyh 2012)
OA	Rat	Knee joint	Not specified (electrophysiological recording)	Sensitization of axon terminals	Pain	(Morgan and others 2022)
OA	Rat	Knee joint	CGRP	Diffuse sprouting	Pain	(Zhang and others 2022)
OA	Rat	Ankle joint)	CGRP	Diffuse sprouting	Pain, sensitization	(Bourassa and others 2020)
OA	Mouse	Knee joint	CGRP, P2X2, PIEZO2	Diffuse sprouting	Pain	(Zhu and others 2019)
OA	Mouse	Knee joint	PGP-9.5, Na_V_1.8	Diffuse sprouting; sensitization and activation of silent nociceptors	Pain	(Obeidat and others 2019)
OA	Mouse	Knee joint	PGP-9.5	Diffuse sprouting	Pain	(Kc and others 2016)
OA	Mouse	Knee joint	CGRP	Diffuse sprouting followed by degeneration of sprouts	Inflammation	(Murakami and others 2015)
OA	Mouse	Knee joint	PIEZO2	Sensitization and activation of silent nociceptors	Pain	(Obeidat and others 2023)
OA	Human	Knee joint	CGRP	Diffuse sprouting	Pain	(Ashraf and others 2011)
OA	Human and rat	Knee joint	CGRP	Diffuse sprouting	Pain	(Aso and others 2020)
OA	Human	Knee joint	SP	Diffuse sprouting and hyperactivity	Inflammation	(Miller and others 2002)
OA	Human	Hip joint	CGRP, SP	Diffuse sprouting	Pain	(Saxler and others 2007)
OA	Human	Knee joint	CGRP, PGP-9.5, SP	Transient denervation	Inflammation	(Eitner and others 2013; Weidler and others 2005)
OA	Human	Knee joint	CGRP, PGP-9.5	Transient denervation of PGP-9.5 profiles but maintained CGRP-positive innervation	Pain	(Ikeuchi and others 2012)
OA	Human	Knee joint	CGRP, PGP-9.5, SP	Sprouting into articular cartilage	Pain	(Suri and others 2007)
RA	Mouse	Ankle joint	CGRP, GAP43	Ramified and diffuse sprouting, formation of neuroma-like elements	Pain	(Gonçalves Dos Santos and others 2020)
RA	Human	Knee joint	SP	Diffuse sprouting	Inflammation	(Miller and others 2002; Miller and others 2000)
RA	Human	Knee joint	SP	Diffuse sprouting followed by transient denervation	Inflammation	(Weidler and others 2005)
RA	Human	Knee joint	SP, PGP-9.5	Initial diffuse sprouting followed by denervation, sensitization, and hyperactivity.	Inflammation	(Miller and others 2000)

CFA = complete Freund’s adjuvant (OA model); CGRP = calcitonin gene-related peptide; GAP43 = growth-associated protein 43; Na_V_1.8 = voltage-gated sodium channel 1.8; NF200 = neurofilament 200; OA = osteoarthritis; P2X2 = purinergic P2X2 receptor; PGP-9.5 = protein gene product 9.5; PIEZO2 = piezo-type mechanosensitive ion channel component 2; RA = rheumatoid arthritis; SP = substance P.

Analysis of CGRP and SP immunoreactive profiles in the human hip joint capsule showed them forming small bundles or running as single fibers mainly in the subintimal part of the synovial layer and to a smaller extent in the fibrous layer of the capsule, forming varicosities and free nerve endings (Laumonerie and others 2021; Saxler and others 2007). In healthy joints, CGRP-positive axons typically outnumber SP profiles, showing an ordered distribution in the surrounding joint vessels or adipose and collagenous tissue (Saxler and others 2007). CGRP profiles were mainly localized along with small arterioles and occasionally at a distance in the fibrocartilage junction of the meniscus, with the inner region of the meniscus typically lacking nociceptive innervations (Ashraf and others 2011). Interestingly, menisci from individuals with severe cartilage damage had more perivascular sensory nerves in the outer region than menisci from individuals with low cartilage damage (Ashraf and others 2011). In OA, the density of CGRP and SP immunoreactive profiles is higher in the joint capsule than in healthy controls (Saxler and others 2007). The increase in sensory innervation in the outer region of the menisci positively correlated with high chondropathy (cartilage damage) scores, with nerve growth in the menisci contributing to OA pain (Ashraf and others 2011). The percentage of osteochondral channels containing CGRP immunoreactive profiles in the knee joint of the symptomatic chondropathy group was higher than in the asymptomatic group. Another study showed that the OA posterior cruciate ligament (PCL) had a density of CGRP-positive profiles similar to that of the control. At the same time, overall nerves immuno-reactive to PGP-9.5 were less in OA PCLs (Ikeuchi and others 2012).

A comparison of the density of SP profiles between synovial tissues of RA and OA joints showed their higher levels in the former (Miller and others 2000). This observation agrees with the notion of SP innervations as proarthritogenic and immunogenic, responding to the increased interleukins (ILs) such as IL-1 and IL-8, cytokines, and tumor necrosis factor α (TNF-α) (Miller and others 2000). Although in the inflamed RA joint, the density of SP axons declines, their overall number is significantly higher when compared to TH-positive fibers (Miller and others 2002), suggesting enhanced activity of peptidergic nociceptors with a likely contribution to the chronic inflammation and pain response.

### Preclinical Studies

**Figure 2.**
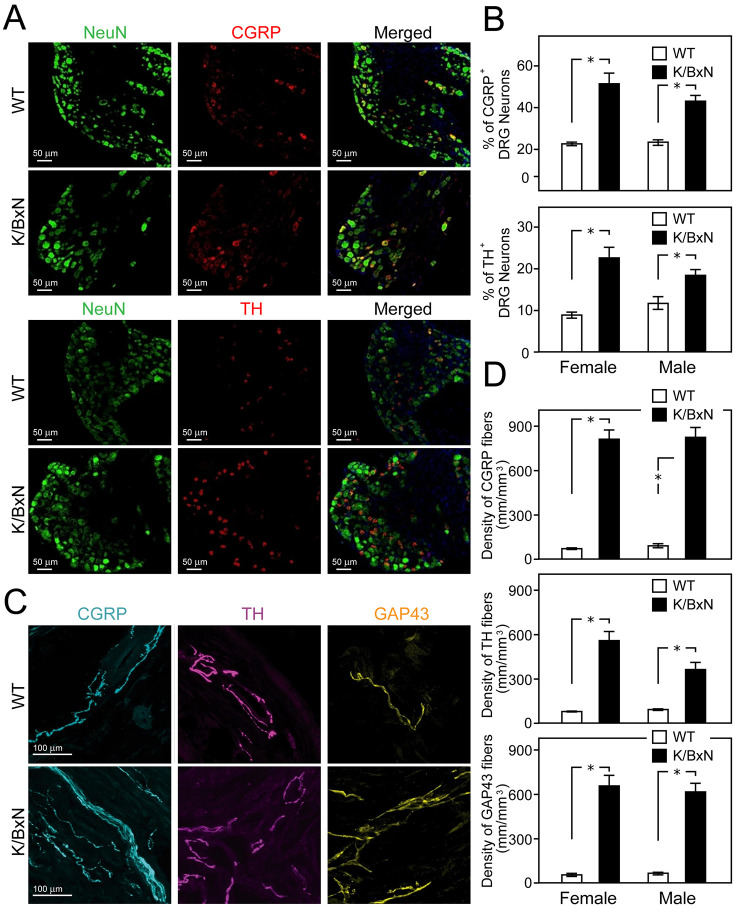
Increase in sensory and sympathetic markers in neurons and the synovial joint of the autoimmune rheumatoid K/BxN model. (A, B) Comparison of calcitonin gene–related peptide (CGRP) and tyrosine hydroxylase (TH) expression in the dorsal root ganglia (DRG) of wild-type and K/BxN mice (16 weeks old). Transgenic mice show an increased number of CGRP^+^ and TH^+^ neurons. DAPI stains nuclei (blue), and NeuN is a general marker for neurons (green). (B) Graphs show the mean percentages ±SEM of NeuN-positive cells counterstained for CGRP (top) and TH (bottom). *P < 0.05. (C, D) Changes in CGRP^+^ and TH^+^ fibers in ankle-joint sections (C) with a summary graphs (D). (C) Representative confocal images of CGRP^+^ (cyan) and TH^+^ (purple) axons counterstained with axon regeneration and plasticity marker GAP43 in wild-type and K/BxN mice joints. Overall, the density of CGRP^+^ and TH^+^ fibers of K/BxN in the synovia is higher than that of WT mice. *P < 0.05. Reproduced with permission from Gonçalves Dos Santos and others (2020).

Several preclinical models have been used to study the response and plasticity of sensory innervations in OA and RA joints, which revealed a range of changes (Table 1). Complete Freund’s adjuvant (CFA) models local inflammation in response to immunogens, mimicking RA. The density of TH and CGRP profiles in the synovial membrane of the CFA model was enhanced, leading to dense arrangements of sensory and autonomic axons and their possible neurochemical cross-talk (Longo and others 2013). The CGRP fibers of the skin over the ankle joint remained unchanged, with functional pain tests showing mechanical and cold allodynia and heat hyperalgesia lasting four weeks postinjection (Longo and others 2013). At 28 days after the CFA injection, a profuse sprouting of CGRP, NF200, TH, and growth-associated protein GAP43 was observed, indicating the formation of new sympathetic and sensory profiles, which were only weakly present in the synovial–meniscal interface in vehicle-treated knee specimens (Jimenez-Andrade and Mantyh 2012). The axonal sprouts showed higher density and disorganized appearance, forming occasional neuroma-like structures (Ghilardi and others 2012). In another rat study, the ankle samples taken four days postinjection showed increased CGRP and NF200 fibers compared to the contralateral control joints (La Hausse De Lalouviere and others 2021). A genetic autoimmune RA K/BxN mouse model expressing both the T-cell receptor (TCR) transgene KRN and the MHC class II molecule A(g7) develops severe inflammatory arthritis owing to higher levels of pathogenic autoantibodies to glucose-6-phosphate isomerase (GPI). Analysis of CGRP and TH expression in dorsal ganglion showed significant enhancement in K/BxN genotypes compared to WT controls (Fig. 2A, B). Higher expression of CGRP, TH, and GAP43 was also observed in axons on the ankle joint samples (Gonçalves Dos Santos and others 2020) (Fig. 2C, D).

**Figure 3.**
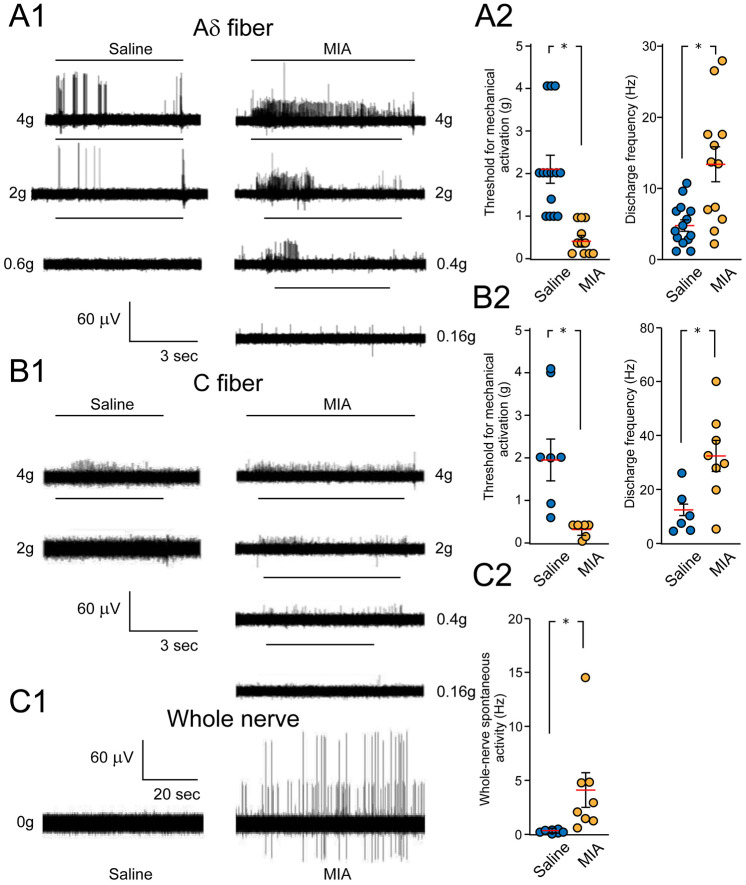
Sensitization of knee joint afferents in a mono-iodoacetate (MIA)–induced osteoarthritis model. (A1, B1) Representative traces of extracellular recordings from Aδ- and C-type nerve fibers of control (saline-injected) and arthritic (MIA-injected) rat joints show an increased response of afferents to von Frey stimulation at various intensities of applied forces (g, grams; black bars above traces). Injections were made 28 days before conducting the electrophysiological recordings. (A2, B2) Summary graphs compare the activation threshold and discharge frequency of Aδ- and C-type nerve fibers in control and MIA groups 3 days and 28 days after inducing the model. (C1, C2) Representative traces of extracellular recordings of whole knee joint nerves show enhanced spontaneous activity in the MIA model relative to saline-injected controls, with respective graphs indicating the group differences. Data represent mean ± SEM. *P < 0.05. Reproduced with permission from Morgan and others (2022).

An increase in the density of sensory fibers in the subchondral bone and synovial membrane was also observed in the monoiodoacetate (MIA) model of OA at 5 weeks and 10 weeks postinjection, with CGRP profiles especially prominent in the subchondral bone, with the enhanced innervations in the synovium and bone correlating with pain behavior (Bourassa and others 2020). In early MIA-induced OA, augmented pain response involves sensitization of nerves in the joint capsule. Extracellular electrophysiological recordings of the knee joint and bone afferents made at day 3 and day 28 in the MIA-induced OA model showed increased response to mechanically evoked activity manifested in a reduction in activation threshold and increased discharge rate at day 3 (Morgan and others 2022) (Fig. 3A, B). Similar recording of the whole nerve also showed enhanced spontaneous spiking activity (Fig. 3C1, C2). Pain response at the later stage of this model, seems to be primarily due to the recruitment of nerves in the subchondral bone (Morgan and others 2022). In the collagenase injection (CIA) model, the density of CGRP-positive fibers in synovial tissue was drastically enhanced, followed by a 35% decline at one week postinjection (Murakami and others 2015). The rate of chondrocyte ossification in chondro- osteophyte lesions of CIA-injected joints correlated with the density of CGRP-positive but not with that of SP-positive profiles (Murakami and others 2015). Notably, SP fiber density in the CIA-injected knee did not change but was twofold higher in the contralateral control knee one to two weeks after injection (Murakami and others 2015). The cause and the mechanisms of increased density of SP-positive profiles in the contralateral joint remain unclear and require further research.

In surgically destabilized medial meniscus (DMM) and *Pkcδ*-null mice models, there was also an increase in nociceptive innervation of the synovium (Kc and others 2016). These changes were correlated with an increase in osteoclasts in early OA, supporting the role of osteoclast-derived netrin 1 in neural remodelling and chronic pain. Immunostaining showed abnormal and dense innervations of CGRP fibers adjacent to the trabecular bone surface one week after surgery, unlike only a few tartrate-resistant acid phosphate (TRAP)–positive osteoclasts and CGRP profiles observed in the control group (Zhu and others 2019). Analysis of a voltage-gated sodium channel, NaV1.8, 16 weeks after destabilized medial meniscus showed no changes in the lateral synovium and cruciate ligaments. In contrast, in the deep layers of the synovium of the medial compartment, the NaV1.8 level was enhanced (Obeidat and others 2019). NaV1.8 expression was also significantly increased in the outer region of the medial meniscus (Obeidat and others 2019). These location-specific discrepancies in the response of NaV1.8 suggest that the molecular rearrangement in OA joints might be exquisitely regulated and involve targeted long-range signalling around the affected tissue.

**Figure 4.**
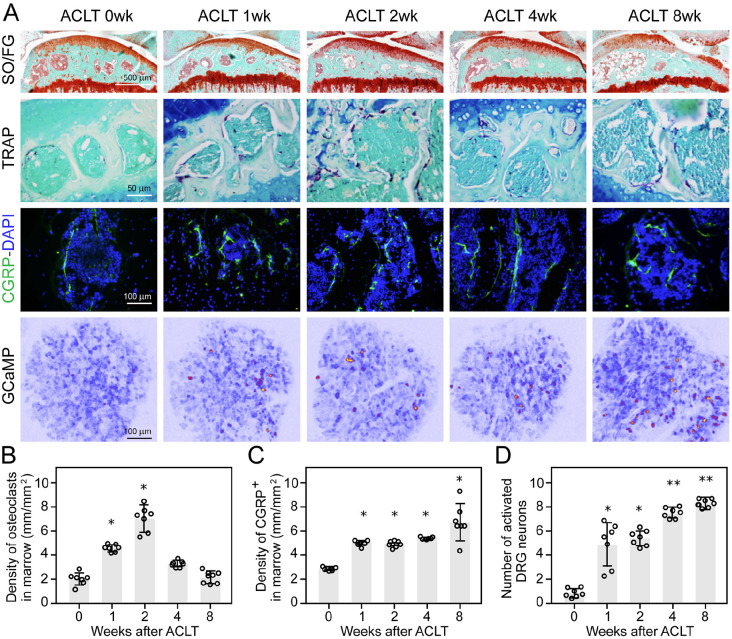
Increase in nociceptive innervations of the OA joint and elevated activity of sensory neurons in the dorsal root ganglia (DRG) of anterior cruciate ligament transection (ACLT) mouse model. (A) Safranin orange/fast green (SO/FG) staining (first row), tartrate-resistant acid phosphate (TRAP) staining (second row, magenta), and immunofluorescence labeling of CGRP^+^ sensory nerve fibers (third row, green counterstained with DAPI, blue) in mouse tibial subchondral bone after ACLT surgery at different time points (wk, weeks). Activity—excitability of respective L4 DRG neurons in vivo in Pirt-GCaMP3 mice in response to mechanical press to the knee (fourth row), measured by imaging calcium dynamics with GCaMP3 reporter at corresponding time points after surgery (as above). Active neurons are labeled in red-yellow. (B–D) Quantitative analysis of the density of TRAP^+^ osteoclasts (B), density of CGRP^+^ sensory nerves in subchondral bone marrow (C), and number of active DRG neurons detected with GCaMP3 recordings (D). *P < 0.05, **P < 0.005 compared with the sham-operated group per each time point. Reproduced with permission from Zhu and others (2019).

In the anterior cruciate ligament transection (ACLT) mouse model of OA, analysis of NF200 A-β fibers and ion channels like P2X2 (purinergic ion channel) and PIEZO2 (mechanosensitive ion channel) showed an increase in both within the subchondral bone marrow. At the same time, the density of NF200-positive fibers remained unchanged (Zhu and others 2019). These results suggest that the innervation of different subgroups of sensory afferents in the subchondral bone of OA is differentially affected. Generation of the ACLT model in *Dmp1-Ranklfl/fl* and *Trap-Ntnfl/fl* transgenic mice showed increased density of CGRP-positive axons in the synovium after OA surgery (Fig. 4A–D). These changes were associated with an increased evoked response of sensory neurons in the dorsal root ganglia (DRG) to mechanical press to the knee, measured by intravital imaging of GCaMP3 activity (Fig. 4A, C). At the same time, the density of CGRP profiles in subchondral bone was decreased (Zhu and others 2019). Analysis of nociceptive axon distribution and response to mechanical stress showed heterogeneity of C-fibers, with increased density of CGRP-positive and other nociceptive nerve endings (P2X3 and PIEZO2), unlike PGP-9.5 density in subchondral bone, which remained unchanged (Zhu and others 2019). In meniscal transection (MNX) OA rat models, the percentage of osteochondral channels containing CGRP immunoreactive profiles was higher than in sham-operated rats (Aso and others 2020). The association of CGRP profile changes with symptoms of pain behavior aligns with the notion that in joints with OA cartilage damage, CGRP-positive axons invade the osteochondral canals, possibly from the bone marrow spaces. Blocking nerve growth factor (NGF) activity by inhibiting its receptor tropomyosin receptor kinase (Trk)–A prevented the growth of CGRP reactive axons, an effect associated with reduced pain behavior, implying that the growth and sprouting of sensory nerves at the osteochondral junction might contribute to chronic OA pain (Aso and others 2020).

Cross-comparison of the knee-joint synovium from three rodent OA models, the ACLT, DMM, and MIA, with normal synovium showed more intense staining for sensory markers and wider sensory marker–positive areas in the DMM OA model (Zhang and others 2022). Analysis of the location of netrin 1, which stimulates the growth and branching of sensory fibers, proved its strong colocalisation with CGRP, suggesting that the increase in sensory innervation in synovium may involve Netrin1. Correlative studies of the relation of CGRP, transient receptor potential vanilloid 1 (TRPV-1), NGF, and netrin 1 to pain sensation showed upregulation in all three OA models (Zhang and others 2022). A recent mouse study showed that knocking out *Piezo2* from nociceptors protected the knockout (KO) mice from mechanical sensitization associated with inflammatory and physical damage–related joint pain, preventing knee swelling and pain induced by intra-articular NGF injection (Obeidat and others 2023). These results suggest that sensitization of joint nociceptors might be mediated by NGF, which is critical for OA pain and depends on the PIEZO2 channel. The process might involve the activation of a unique subset of joint axons known as “silent nociceptors,” which could contribute to enhanced pain response (Obeidat and others 2023).

## Remodelling Autonomic Innervations in Inflamed Synovial Joints

Alterations of autonomic innervations in synovial joints and related neuropathy have also been implicated in the pathobiology and manifestation of RA and OA. Clinical and preclinical studies indicate changes in the density and distribution of sympathetic and parasympathetic profiles. Unlike alterations in nociceptive innervations with overall proinflammatory effects, autonomic innervations and activity impairments can produce either anti- or proinflammatory effects. In general, alterations in autonomic drive in RA joints are proinflammatory, whereas in OA, the outcomes are anti-inflammatory.

## Sympathetic Innervation of Inflamed Joints

### Clinical Studies

Since the 1950s, it has been well-known that sympathetic innervations influence the inflammatory response, including in synovial joints (Capellino and others 2010; Jänig 2006; Korner and others 2019). Unlike nociceptive profiles immunoreactive to antibodies against SP, CGRP, TRPV1, PIEZO1/2, and acid-sensing ion channels (ASIC), the postganglionic sympathetic axons stain positive for TH and VMAT-2. In OA human joints, articular cartilage contains CPON-positive sympathetic nerves (Suri and others 2007). In synovial joints of patients with RA, sympathetic profiles show a marked pruning, contrasting with the extensive sprouting of sensory fibers (Weidler and others 2005). The loss of sympathetic innervations in RA correlates with reduced local inflammation and alters the ratio of sensory to sympathetic profiles in affected joints. Notably, the response of autonomic innervation in RA and OA differs, with TH immunoreactive profiles lower in the former (Weidler and others 2005). Accordingly, the ratio of sympathetic to sensory fibers in RA is 1:5, while in OA, it is 1.5:1, favoring the local inflammatory process in the former (Miller and others 2002). The mechanisms underlying the differential response of sympathetic innervations to RA versus OA remain a subject of future analysis in patients and preclinical models.

Higher sympathetic drive within synovial joints, in addition to favoring the release of proinflammatory factors from local cells, also can promote plasma extravasation and a decrease in vascular tone, facilitating the invasion of immune cells into the affected area. Notably, the loss of sympathetic innervation of the RA joint, in addition to local anti-inflammatory effects, also interrupts the sympathetic influence of the central hypothalamus–autonomic regulatory axis on the joint (Miller and others 2000) (Fig. 1). It must be noted that despite the loss of sympathetic profiles in RA and OA joints, the amount of NE release and its activity level remain relatively high (Capellino and others 2010). This paradoxical effect can be attributed to the appearance of numerous local TH immunoreactive cells. The absence of these cells in healthy joints suggests the compensatory nature of the response (Capellino and others 2010). Among cells that could produce catecholamines, fibroblasts, macrophages, B cells, mast cells, and granulocytes have been considered, with their catecholaminergic effects reported to be triggered by local inflammation. Whether NE released by these TH-positive cells impacts the local inflammatory response in arthritic joints remains to be shown.

### Preclinical Studies

A remodelling of sympathetic nerve fibers in joints has also been reported in preclinical models of OA and RA (see Table 2) (Fig. 2). Unlike normal synovial joints where sympathetic fibers align with blood vessels in the lower dermis, in the CIA-induced RA joints of mice, the TH-positive fibers wrap around peptidergic nociceptive profiles in the synovial membrane, suggesting their involvement in pain and neuroinflammation response (Longo and others 2013). In CFA-injected joints, the density of sympathetic VMAT-positive profiles was enhanced. The ectopic presence of sympathetic fibers in the upper dermis was detectable two weeks post-CFA injection, with their density in the dermis and synovial membrane increasing over four weeks after inducing the model. Like CGRP and NF200 sensory axons, TH-positive sympathetic fibers undergo sprouting, as indicated by the enhanced presence of GAP43 in affected joints (Jimenez-Andrade and others 2012; Longo and others 2013). At 28 days after the CFA injection, sympathetic and nociceptive nerve fiber sprouting was also observed within the joint synovium. The newly sprouted nerve fibres are present at significantly higher density and display a disorganised appearance compared to their regular distribution in the synovium of vehicle-treated or naïve mice. The density of GAP43 immunoreactive profiles undergoing sprouting was also increased in the femoral periosteum of mice receiving intra-articular CFA injections (Ghilardi and others 2012; Jimenez-Andrade and others 2012). Analysis of the effect of the sympathetic drive on inflammation of CIA joints showed its bimodal character, with time dependent enhancement or suppression of pro-inflammatory and anti-inflammatory cytokines. The direction of the response depends on the time of immune system activation, specific compartment of the joint, and affected tissue type (Härle and others 2005). It emerges that the sympathetic effects are proinflammatory during the asymptomatic phase of CIA, whereas during the symptomatic phase, they become anti-inflammatory. Accordingly, sympathectomy of the joint before injection of collagen II markedly decreased the severity of arthritis (Härle and others 2005).

**Table 2.** Alterations in Sympathetic Innervation of Arthritic Joints in Human Studies and Preclinical Models, with Analyzed Joints, Specific Changes in Innervations, Functional Readouts, and References.

Condition	Model	Joint	Axonal markers	Remodelling and plasticity	Readouts	References
OA	Rat	Ankle joint	VMAT-2	Ectopic sprouting, entwining with sensory fibers	Pain, sensitization	(Longo and others 2013)
OA	Mouse	Knee joint	TH, GAP43	Ectopic and disorganized sprouting	Pain	(Ghilardi and others 2012)
OA	Mouse	Knee joint	TH, GAP43	Ectopic and disorganized sprouting, neuroma-like elements	Pain, inflammation	(Jimenez-Andrade and others 2012)
OA	Rat	Ankle joint	VMAT-2	Ectopic sprouting, entwining with sensory fibers	Pain, sensitization	(Bourassa and others 2020)
OA	Rat	Temporomandibular joint	TH	Ectopic sprouting	Joint deformation	(Jiao and others 2015)
OA	Human	Knee joint	TH, PGP-9.5	Denervation	Inflammation	(Eitner and others 2013)
OA	Human	Knee joint	CPON, PGP-9.5	Innervation of articular cartilage	Pain	(Suri and others 2007)
RA	Mouse	Ankle joint	TH, GAP43	Ectopic and disorganized sprouting, neuroma-like elements	Pain	(Gonçalves Dos Santos and others 2020)
RA	Mouse	Ankle joint	TH, VMAT-2	Denervation, stage-dependent pro- and anti-inflammatory effects	Inflammation	(Capellino and others 2010)
RA	Mouse	Knee joint	TH	Denervation, activation of sensory fibers, stage-dependent pro- and anti-inflammatory effects	Inflammation, pain	(Härle and others 2005)
RA	Human	Knee joint	TH	Denervation	Inflammation	(Miller and others 2002)
RA, OA	Human	Knee joint	TH	Denervation; Appearance of TH-positive profiles; pro- and anti-inflammatory effects	Inflammation	(Capellino and others 2010)
RA, OA	Human	Knee joint	TH	Diffuse sprouting (OA) Nondiscriminatory denervation (RA)	Inflammation	(Weidler and others 2005)
RA	Human	Knee joint	Neuropilin 2	Denervation	Inflammation	(Fassold and others 2009)
RA	Human	Knee joint	TH	Denervation	Inflammation	(Miller and others 2000)

CFA = complete Freund’s adjuvant; CPON = C-flanking peptide of neuropeptide Y; GAP43 = growth-associated protein 43; Neuropilin 2 = a nerve repellent receptor; OA, osteoarthritis; PGP-9.5 = protein gene product 9.5; RA, rheumatoid arthritis; TH = tyrosine hydroxylase; VMAT-2 = vesicular monoamine transporter 2.

In OA models, injury caused by excessive mechanical loading in rodent temporomandibular joint (TMJ) through dental occlusion led to the sprouting of sympathetic nerve fibers of the subchondral bone (Jiao and others 2015). The level of NE in subchondral bone increased significantly at four and eight weeks of experimental groups compared to age-matched controls. Profuse sprouting of sympathetic nerve fibers and increased β2-adrenergic receptor (Adrb2) mRNA expression, as well as the presence of Adrb2 immunopositive cells, were observed in the condylar of the subchondral bone of the four-week experimental groups (Jiao and others 2015). In the same vein, in the MIA rat ankle model of OA, VMAT-2 and CGRP nerve fibers sprouted outside the haversian canals, leading to profuse innervations of subchondral bone and synovial membrane at 5 and 10 weeks post-injection (Bourassa and others 2020). The intense sprouting of sympathetic fibers leads to high density of innervations with close juxtaposition of sensory and sympathetic profiles in affected areas. Functional studies of sensory–sympathetic interactions show that sympathetic effects on sensory innervations are excitatory (Härle and others 2005), with the increase in sympathetic drive causing C-fiber hyperexcitability, contributing to their sensitization. An increase in the density of sympathetic fibers has also been observed in the autoimmune inflammatory K/BxN RA mouse model (Gonçalves Dos Santos and others 2020) (Fig. 2C, D). Unlike well-structured sympathetic profiles in controls, in K/BxN RA mice, most TH and GAP43 immunoreactive sprouts show a disorganized distribution. These findings infer an extensive remodelling of TH-positive innervations in arthritic joints, which is likely to contribute to the sensitization of nociceptive axons in the area and promote their spontaneous discharge activity.

## Parasympathetic Remodelling in Inflamed Joints

### Clinical studies

**Figure 5.**
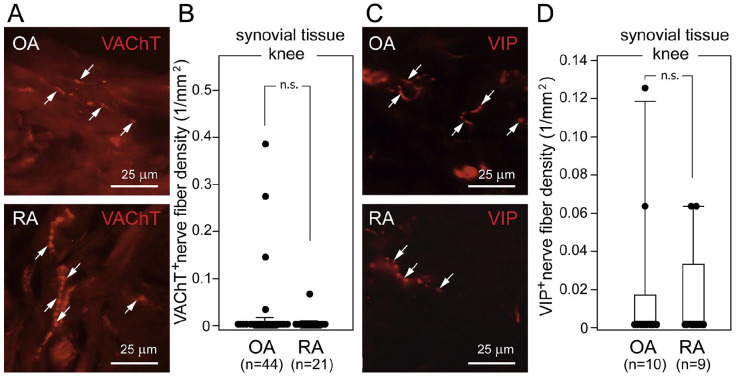
Parasympathetic nerve fibers in knee synovial tissue of patients with osteoarthritis (OA) and rheumatoid arthritis (RA). (A, C) Representative micrographs of cholinergic VAChT-positive nerve fibers (A, arrows in upper and lower image) and cholinergic VIP-positive nerve fibers (C, arrows in upper and lower image) in synovial knee tissue from patients with OA or RA (top and bottom, respectively) undergoing total knee joint replacement surgery. (B) Comparison of the density of cholinergic VAChT-positive nerve fibers in synovial knee tissue from patients with OA and RA. (D) Comparison of cholinergic VIP-positive nerve fibers in synovial knee tissue from patients with OA and RA. One dot indicates one patient’s mean nerve fiber density in synovial tissue. Box plots demonstrate the 10th (whisker), 25th, 50th (median), 75th, and 90th (whisker) percentile. n.s. = not significant; VAChT = vesicular acetylcholine transporter; VIP = vasoactive intestinal peptide. Reproduced with permission from Stangl and others (2015).

Although recognized primarily as a disease affecting synovial joints, RA effects extend over other organs and systems, manifesting through general symptoms such as tiredness, weight loss, and disruptions of autonomic functions (Conforti and others 2021; Saba and Sultan 2014). Table 3 summarizes arthritis-related alterations of general parasympathetic activity and local changes in synovial joints. Analysis of heart rate showed a reduction of parasympathetic effects in patients with RA compared to controls, with individuals at risk of RA also showing slower heart rates. Interestingly, there seems to be a positive correlation between changes in parasympathetic effects and pain intensity in affected joints (Geenen and others 1996). A deficit in parasympathetic drive with reduced vagal activity in RA is thought to favor systemic and local inflammation in affected joints (Altawil and others 2012). In healthy humans, no VAChT or VIP immunoreactive cholinergic profiles have been observed in synovial tissue (Stangl and others 2015). In contrast, in synovial tissue from finger joints of individuals with OA and RA, some VAChT and VIP immunoreactive nerve fibers have been detected, with their number correlating with the extent of inflammation (Stangl and others 2015). VAChT and VIP immunoreactive fibers were also detected in synovial tissue of the knee of patients with OA and RA, with their density closely matching between the two groups (Fig. 5A–D). It should be noted that in highly inflamed lesions of RA synovial joints, VAChT-positive profiles, like TH immunoreactive fibers, undergo extensive degeneration. A similar trend has also been observed in the RA and OA periosteum and joint tissue adjacent to arthritic inflammation (Courties and others 2017; Stangl and others 2015). There has been some evidence suggesting that VAChT and VIP immunoreactive axons might be derived from TH-positive fibers through their reprogramming (see below). Although the mechanisms underlying the phenotypic switch of fibers of arthritic joints remain to be established, gp130-signalling leukemia inhibitory factor (LIF), as well as several cytokines and extracellular matrix proteins, have been implicated (Stangl and others 2015).

**Table 3.** Alterations in Parasympathetic Activity and Innervation of Arthritic Joints in Human Studies and in Preclinical Models, with Analyzed Joints, Specific Changes in Innervations, Functional Readouts, and References.

Condition	Model	Joint	Axonal Markers	Remodelling and Plasticity	Readouts	References
RA	Human	Not specified	Not specified (HRV)	Reduction in vagal function	Pain	(Geenen and others 1996)
RA	Human	Not specified	Not specified (HR changes)	Reduction in vagal function	Inflammation	(Altawil and others 2012)
RA, OA	Human	Knee and finger joints	VAChT, VIP	Lower density of cholinergic fibers in RA synovium vs. OA synovium; phenotypic switch of sympathetic to parasympathetic innervations	Inflammation	(Stangl and others 2015)
RA	Mouse	Ankle joint	VAChT	Increase in cholinergic innervations compared to catecholaminergic fibers; phenotypic switch of sympathetic to parasympathetic innervations	Inflammation	(Stangl and others 2015)

HRV = heart rate variability; HR = heart rate; OA = osteoarthritis; RA = rheumatoid arthritis; VAChT; vesicular acetylcholine transporter; VIP = vasoactive intestinal protein.

### Preclinical Studies

The results of animal studies of cholinergic innervations in arthritic joints generally agree with clinical findings in humans. In control rats, cholinergic profiles are confined to the skin or muscle tissue surrounding synovial joints (Stangl and others 2015). In the CIA model of arthritis, the response of cholinergic profiles differs from TH-positive fibers (Stangl and others 2015). Unlike the depletion of TH immunoreactive innervations of joints at 35 days post-CIA injection, the density of VAChT reactive parasympathetic fibers is significantly higher (Stangl and others 2015). The resulting 3:1 ratio of VAChT/TH fibers calculated for individual limbs of mice was the highest on 35 days postimmunization, followed by a decline to the initial levels on day 55. The reasons for the transient and selective rise in cholinergic innervations remain unclear and could reflect compensatory adjustments of the parasympathetic versus sympathetic drives-to tone down the local inflammation and favor the recovery processes. Notably, the focal bony erosions were almost devoid of cholinergic nerve fibers, with very few VAChT-positive profiles detected in inflamed regions surrounding erosions, which possibly originated from cholinergic axons of the periosteum (Stangl and others 2015).

## Mechanistic Considerations for Neuroplasticity in the Arthritic Joint

Our review of preclinical and clinical studies shows that sensory and autonomic innervations in arthritic joints undergo extensive remodelling, which may contribute to neuroinflammation and pain. Several signalling pathways are likely at play, regulated by an array of trophic and growth factors and inflammatory mediators, determining the type of changes (Wood and others 2022; Zhu and others 2019).

**Figure 6.**
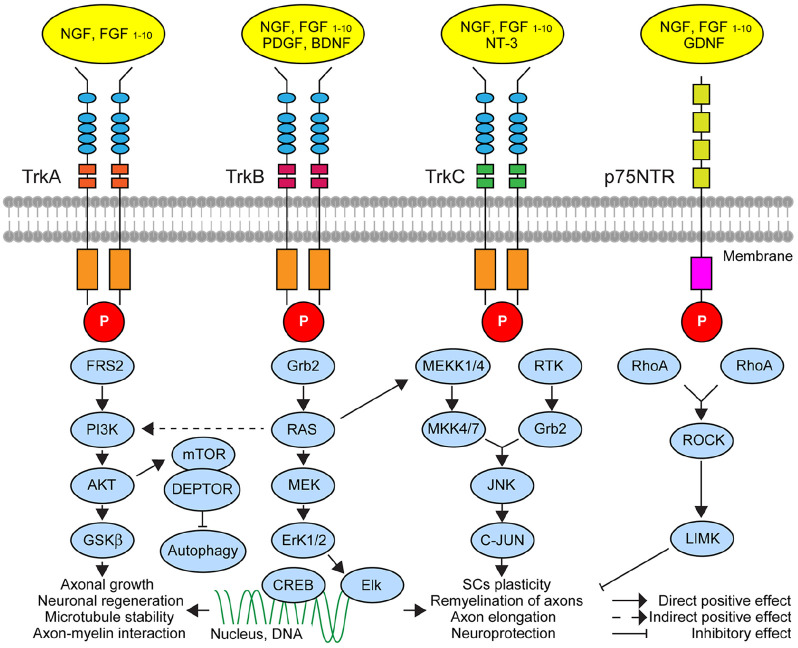
A simplified schematic of major neurotrophins and their signalling mechanisms and targets implicated in axonal growth and plasticity that might contribute to the neuroinflammation and pain response in rheumatoid arthritis (RA) and osteoarthritis (OA). Binding trophic factors to their receptors (four primary receptors, TrkA, TrkB, TrkC, and p75NTR) at axon terminals leads to activation of molecular cascades with downstream effects on axonal outgrowth or regeneration, microtubule stability, and neuronal survival. These mechanisms may involve long-range retro-axonal signalling with an impact on the genome of sensory neurons in dorsal root ganglia and sympathetic neurons in sympathetic ganglion (TrkA, TrkB, and TrkC), respectively, or local effects through modulation of molecular processes at the site of action (p75NTR). The initiation of the response involves phosphorylation of the membrane receptors, which regulates molecular processes through different adaptor proteins via direct and indirect effects. Some key players in neurotrophic signalling pathways are currently explored as therapeutic targets for OA and RA. AKT = AKT serine/threonine-protein kinases; BDNF = brain-derived neurotrophic factor; CREB = the cAMP-responsive element-binding protein; Elk = ETS-like 1 protein; Erk1/2 = extracellular signal-regulated kinases 1/2; FGF = fibroblast growth factor; GDNF = glial-derived neurotrophic factor; Grb2 = growth factor receptor-bound protein 2; GSKb = glycogen synthase kinase 3; JNK = c-Jun N-terminal kinases; LIMK = LIM domain kinase 1; MEK = mitogen-activated protein kinase; MEKK1/4 = mitogen-activated protein kinase kinase 1/4; mTOR = mammalian target rapamycin protein; NGF = nerve growth factor; NT3 = neurotrophin 3; PDGF = platelet-derived growth factor; PIK3 = phosphatidylinositol-4,5-bisphosphate 3-kinase; RAS = Ras proteins; RhoA = RhoA protein; ROCK = Rho-associated protein kinase; RTK = receptor tyrosine kinases; Tkr = tyrosine receptor kinase. For further details, the readers are referred to reviews of neurotrophic signalling in axonal growth, stability, and plasticity (Kashyap and others 2018; Li and others 2020a).

Among trophic and growth factors promoting neurite elongation and sprouting, NGF, brain-derived neurotrophic factor (BDNF), neurotrophins (NTs), fibroblast growth factor (FGF), and glia-derived growth factor (GDNF) have been most widely considered (Hromadkova and others 2020; Isaacson and others 1992; Kalil and Dent 2014; Li and others 2020a; Weidler and others 2005) (Fig. 6). While several reports presented evidence supporting the role of NGF in neural remodelling and plasticity in arthritic joints (Aso and others 2020; Obeidat and others 2023), the direct involvement of others remains to be shown. It is important to note that enhanced NGF-TrkA signalling has also been implicated in the hyperactivity of nociceptors through promoting the synthesis and release of pain-mediating peptides (SP, CGRP, VIP, bradykinin) as well as via changes in the expression and activity of neurotransmitter receptors and ion channels (Barker and others 2020; Ghilardi and others 2012; Malcangio and others 1997; Ye and others 2018).

Elevated NGF in chronic inflammation and associated joint damage can also trigger the sprouting of autonomic axons, enhancing the sympathetic innervation of joint tissue and lower dermis (Dietz and others 2021; Longo and others 2013; Shang and others 2017). Interestingly, unlike NGF acting equally on sensory and autonomic axons of OA joints, the level of BDNF expression correlated with the increase of TH-positive sympathetic but not SP-reactive profiles (Ashraf and others 2011). The unchanged BDNF expression in the RA joints of patients suggests its specific role in OA, where TrkB activation promotes the growth of sympathetic nerve fibers but with little effect on nociceptive axons (Ashraf and others 2011). Finally, Stangl and coworkers suggested that the autonomic innervation of joints can be regulated by progesterone controlling the expression of the VAChT gene (SLC18A3) acting upon the hormone-receptor binding element site (Stangl and others 2015). Accordingly, higher progesterone activity was shown to enhance the expression of cholinergic markers and lower the expression of TH in affected tissue (Stangl and others 2015).

Retraction, atrophy, and degeneration of sensory and autonomic axons observed in OA and RA also play a critical role in the rewiring of arthritic joints, with evidence inferring the involvement of semaphorins (Damo and Simonetti 2022; Igea and others 2022; Li and others 2020b). In arthritic joints, these versatile nerve repellents can activate neurite pruning and apoptosis and inhibit axonal growth (Alto and Terman 2017; Miller and others 2000). Accordingly, the expression level and activity of semaphorins are shown to rise in inflamed joints of patients with RA and OA, acting upon sensory and autonomic fibers (Miller and others 2000). Increased levels of semaphorin 3C in RA joints could be the principal determinant of reduced density of sympathetic nerve fibers compared to controls and OA patients (Weidler and others 2005). The retraction of neurites to semaphorins is mediated by neuropilin 2 and plexin A2 receptors, which, upon activation, enhances sympathetic fiber repulsion and lead to their degeneration (Fassold and others 2009).

Another major player implicated in retraction and the loss of sympathetic nerve fibers in arthritic joints is local high concentrations of NGF (Miller and others 2000; Scott-Solomon and others 2021), which can activate the low-affinity neurotrophic receptor p75 and induce axon withdrawal and neurodegeneration (Becker and others 2018; Ovsepian and Herms 2013; Pathak and others 2018). This mechanism plays an essential role in pruning of axons during normal development and can contribute to tissue damage and apoptosis under stress, injury, and neurodegenerative diseases (Ibanez and Simi 2012; Kraemer and others 2014; Ovsepian and others 2016).

## Limitations of Reviewed Studies

Presented herein evidence for neural remodelling and plasticity in arthritis should be treated with a degree of caution, considering three potential limitations of the analyzed reports. First, nearly all described data are based on histopathological and immunofluorescence analysis in fixed tissue; therefore, they are prone to errors introduced by sample preparation, specificity of antibodies, and possible cross-reactivity. Second, although preclinical studies in animal models replicate the neurobiological alterations found in joints of patients with OA and RA, the models present only specific aspects of the pathology, subject to the effects of interspecies differences between animal systems and humans. Finally, most of the physiological inferences are based on changes in molecular markers and, as such, are correlative and warrant direct functional verifications using electrophysiological and live imaging analysis. Future studies should consider these limitations to improve the quality and accuracy of observations to set a firmer ground for drawing assumptions and making therapeutic projections.

## Conclusions and Future Directions

**Figure 7.**
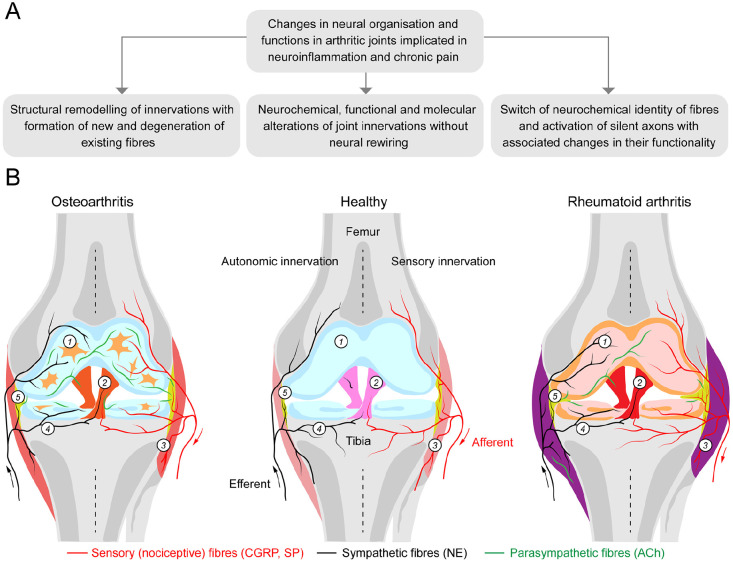
Alterations in sensory and autonomic innervations in arthritic joints implicated in neuroinflammation and chronic pain of osteoarthritis (OA) and rheumatoid arthritis (RA). (A) Three sets of changes in neural wiring and function in arthritic joints in preclinical and clinical studies of RA and OA. (B) Schematic illustration of sensory and autonomic innervations in healthy, osteoarthritis, and rheumatoid arthritis joints. A healthy knee joint has a smooth articular cartilage (1) covering the underlying subchondral bone, (4) surrounded by a joint capsule and (3) coated by a thin layer of synovial tissue (5). The meniscus, a fibrocartilage tissue, acts as a cushion, preventing bones from rubbing against each other. Ligaments (2) provide the joint with mechanical stability. Sensory and sympathetic nerves are observed within the synovium, bone, outer region of the meniscus, and the joint capsule. In OA, the density of sensory (substance P) and sympathetic (tyrosine hydroxylase) nerve fibers decreases in the synovium, with the ratio of sympathetic to sensory profiles reported as 1.5:1; both sensory and sympathetic nerve fibers become prevalent (in equal proportions) in the articular cartilage and surrounding tissue. In RA, the density of sensory fibers is enhanced in the synovium, whereas the density of sympathetic profiles decreases, with the ratio of sympathetic to sensory profiles 1:5. Parasympathetic nerve fibers expressing vesicular acetylcholine transporter (VAChT) and vasoactive intestinal peptide (VIP), absent in healthy joints, become visible in RA- and OA-affected joints (not shown in the figure) with higher densities in OA than in RA. The origin of newly formed parasympathetic profiles remains a subject for future research.

Inflammation and pain in synovial joints are principal hallmarks of OA and RA, the most common form of arthritis, affecting over 10 million people in the United Kingdom alone. Despite impressive progress in research and improving therapies, the neurobiological mechanisms underlying arthritic pain and inflammation warrant further studies to develop more effective and targeted interventions. This review has focused on the remodelling and plasticity of sensory and autonomic innervations of joints in clinical and preclinical studies, which contribute to neuroinflammation and chronic pain. From the literature analysis, three sets of changes have emerged: (1) molecular and neurochemical alterations in existing sensory and autonomic profiles, (2) structural remodelling of established innervations with the formation of new and loss of existing fibers and (3) change of the neurochemical identity of autonomic profiles and activation of silent nociceptors (Fig. 7). While sensitization of C-type nociceptive afferents leading to their hyperactivity has been viewed as the principal underpinning of neuroinflammation and pain in joints, the evidence presented herein also suggests the critical importance of autonomic drive with remodelling of sympathetic and parasympathetic innervations.

In addition to affording critical mechanistic insights into the pathobiology of OA and RA, described herein alterations also suggest potential roadmaps for developing therapies aiming at (1) reducing the release of pain peptides and mediators of inflammation, to restore the neurochemical balance in arthritic joints; (2) preventing the neural rewiring and development of ectopic axon collaterals or their loss and (3) averting the reprogramming and switch of axon types, to counter the neuroinflammation and restore the neurochemical homeostasis. Much progress has been made recently in curbing the overactive nociceptors by inhibiting the release of pain peptides using therapeutic botulinum toxin type A (BoNT/A) and gene therapies targeting the activity of peripheral nerves (Blanshan and Krug 2020; Goins and others 2012; Nguyen and others 2022; Ovsepian and others 2019; Ovsepian and Waxman 2023; Singh 2010). The focus of the efforts to prevent rewiring and sprouting, on the other hand, has been on the therapeutic use of trophic factors and modulation of their signalling and targets, with several promising discoveries in preclinical studies entering clinical trials (Malfait and others 2020; Schmelz and others 2019; von Loga and others 2019; Zhao and others 2024). Although both research directions have yielded exciting results, the translational challenges and risks of adverse effects merit further studies to improve the selectivity and safety of interventions. Given the rapidly expanding molecular targets and emerging methods for precision medicine, we anticipate substantial progress in developing effective and personalized treatments for OA and RA in the foreseeable future.
